# Tea Consumption Enhances Endothelial-Dependent Vasodilation; a Meta-Analysis

**DOI:** 10.1371/journal.pone.0016974

**Published:** 2011-03-04

**Authors:** Rouyanne T. Ras, Peter L. Zock, Richard Draijer

**Affiliations:** Nutrition and Health Department, Unilever R&D Vlaardingen, Vlaardingen, The Netherlands; Copenhagen University Hospital Gentofte, Denmark

## Abstract

**Background:**

Tea consumption is associated with a lower risk of cardiovascular disease including stroke. Direct effects of tea components on the vasculature, particularly the endothelium, may partly explain this association.

**Objective:**

We performed a meta-analysis of controlled human intervention studies on the effect of tea on flow-mediated dilation (FMD) of the brachial artery, a measurement of endothelial function, which is suggested to be associated with cardiovascular risk.

**Methods:**

Human intervention studies were identified by systematic search of the databases Medline, Embase, Chemical Abstracts and Biosis through March 2009 and by hand-searching related articles. Studies were selected based on predefined criteria: intervention with tea as the sole experimental variable, placebo-controlled design, and no missing data on FMD outcome or its variability. A random effects model was used to calculate the pooled overall effect on FMD due to the intake of tea. The impact of various subject and treatment characteristics was investigated in the presence of heterogeneity.

**Results:**

In total, 9 studies from different research groups were included with 15 relevant study arms. The overall absolute increase in FMD of tea vs. placebo was 2.6% of the arterial diameter (95% CI: 1.8-3.3%; P-value <0.001) for a median daily dose of 500 mL of tea (2–3 cups). This is a relative increase of approximately 40% compared to the average FMD of 6.3% measured under placebo or baseline conditions. There was significant heterogeneity between studies (P-value <0.001) that might partly be explained by the cuff position either distal or proximal to the area of FMD measurement. No indication for publication bias was found.

**Conclusion:**

Moderate consumption of tea substantially enhances endothelial-dependent vasodilation. This may provide a mechanistic explanation for the reduced risk of cardiovascular events and stroke observed among tea drinkers.

## Introduction

Tea consumption is associated with a reduced risk for cardiovascular disease (CVD) including stroke [1]–[3]. A suggested mechanism that may partly explain this association is the direct effect of tea on the vasculature, particularly the endothelium [3]. The endothelium, the inner lining of all blood vessels, plays a central role in vascular homeostasis, including maintenance of vascular tone, balancing blood fluidity and blood clotting, and vascular inflammatory processes [4]. Endothelial dysfunction refers to disruption of the homeostatic endothelial condition, and plays an important role in the development of atherosclerosis and CVD [5]. Many CVD risk factors such as hypercholesterolemia, diabetes, hypertension, ageing, smoking and hyperglycaemia, have been shown to be accompanied by endothelial dysfunction [6], [7].

Endothelial (dys)function can be determined by assessing the degree of flow-mediated dilation (FMD) of the brachial artery [8], [9]. FMD represents the endothelium-dependent relaxation of the artery, mediated via release of nitric oxide (NO), in response to a hyperaemic stimulus (increased flow established by the release of a supra-systolic inflated cuff around the arm), and is seen as a direct and reliable measure of vascular reactivity of the macro circulatory system. Although not conclusive [10]–[12], evidence from prospective studies suggests that FMD is independently inversely associated with cardiovascular events [13]–[18].

Several human intervention studies have investigated the effect of tea consumption on endothelial function [19]–[26]. The majority of these studies report a beneficial effect of tea on FMD. A meta-analysis by Hooper et al. [27] on flavonoids/flavonoid-rich foods and cardiovascular risk factors suggested a beneficial effect of black tea on FMD of 3.40% (95%CI: 1.85–4.95%) after long-term consumption, and 1.70% (95%CI: -0.17-3.57%) acutely after tea intake. These data were, however, based on only 2 studies, and several other studies on black and green tea and FMD have been published since.

We performed a meta-analysis of currently available data from human intervention studies on the effect of tea consumption on endothelial function as measured by FMD of the brachial artery. Objectives were to estimate the size of the overall effect and to identify the impact of various subject and treatment characteristics on the effect of tea on FMD in the presence of heterogeneity.

## Methods

The supporting PRISMA checklist for this meta-analysis is available as supporting information; see Checklist S1.

### Search strategy

Potential relevant published studies were identified from the databases Medline, Embase, Chemical Abstracts and Biosis (from starting date of the databases until March 2009). The following search terms were used to search in titles and abstracts: (tea or black tea or green tea or flavonoid? or tea extract? or tea component? or tea solid? or camellia sinensis) and (flow-mediated or flow mediated or FMD or endothelial function or endothelial dysfunction or endothelium-dependent or blood flow or arterial stiffness or vascular resistance or circulat* or micro-circulat* or microcirculat* or vasodilat* or dilat*). The search was limited to studies in human adults.

### Selection criteria

We selected human intervention studies that investigated the relationship between tea intake and FMD. The selection was performed in 2 steps. The first selection step was based on titles and abstracts. Studies were included if they met the following inclusion criteria: human intervention study with adults (with parallel or crossover design), intervention with tea as the only experimental variable, outcomes related to vascular function, endothelial function or FMD and no intentional co-intervention from which the effect of tea could not be isolated. During the second selection step, full texts of the papers were read to check whether the first selection step was done correctly, and to exclude studies based on the following criteria: missing data on FMD, no measures of variability of FMD reported, no suitable control treatment included in the design, and no full text available. We focussed on studies with freshly brewed tea or with tea powder that was produced by drying freshly brewed tea, excluding purified or isolated substances from tea. In case of unclarity, inclusion of a study was discussed among 2 of the authors (RTR and RD) to reach a decision. For completeness, potential relevant and eligible studies published after completion of the systematic search through March 2009 were also included.

### Data extraction and quality assessment

For each of the studies selected, data were extracted using a custom-made database on identification of the study (author, year of publication, country), study design (parallel or crossover), subject characteristics (age, body mass index (BMI), gender, ethnicity and health status), diet, treatment characteristics (green or black tea, dosage of tea, duration, preparation of tea), FMD analysis characteristics (fasted or non-fasted, position of cuff, time of occlusion), FMD values with accompanying measures of variance, and study quality. When data were missing, the authors of the original study were approached to obtain these data (successful in 2 occasions [24], [28]).

Quality of the studies was assessed by to a tool that was specifically developed for this meta-analysis based on the Delphi Consensus [29]. The following criteria were used for scoring the quality of each study: proper randomization procedure (quality score  = 1 point), similarity of treatment groups in relevant variables at baseline (1 point), specification of eligibility criteria (1 point), blinding of subjects and investigators (each 1 point), valid point estimates and measures of variability presented for FMD (1 point), and data on degree of compliance (1 point). A combined quality score was obtained by adding the scores for each criterion. Thus, quality score could range from 0 to 7 points. The quality scores were used only for performing subgroup analyses to examine whether the net response in FMD was different in studies with high vs. low quality. Because scoring of quality is intrinsically subjective, the quality scores were not used to exclude lower quality studies from the meta-analysis or to weigh the studies.

### Statistical analysis

FMD is expressed as follows: FMD (%)  =  [(hyperaemic diameter – resting diameter)/resting diameter] ×100 [8], where resting diameter is the diameter of the brachial artery before any flow stimulus in the artery is created, and hyperaemic diameter is the diameter of the artery observed after release of the inflated cuff. For cross-over studies, the net response in FMD was calculated by subtracting the mean FMD value at the end of control period from the mean FMD value at the end of the treatment period. For parallel studies, the net response in FMD was calculated by subtracting the mean change in FMD in the control group from the mean change in the treatment group, where mean change is FMD value at the end of the intervention minus the FMD value at the start of the intervention.

For calculating the pooled overall effect of tea on FMD, we weighed the studies by the inverse of their variance (1/SE^2^) (SE  =  standard error), giving more weight to more precise studies. When not provided, the SE of the net change was derived from P-values or calculated according to the equations by Follmann et al. [30]. For the latter, we assumed a within-subject correlation coefficient of 0.5 [31]. Since it is unlikely that all the heterogeneity in results is due to the treatment itself, a random effect model according to the methods described by DerSimonian and Laird and van Houwelingen et al. was used to take both within- and between-trial variance into account [32], [33]. Calculations were done using the PROC MIXED model of the SAS analytical system (version 9.2).

The extent of heterogeneity between studies was assessed by estimating the proportion of total variation across studies due to variability between studies rather than due to chance, using the Cochran's Q statistic and the I^2^ statistic [32], [34], [35]. The I^2^-statistic ranges between 0 and 100%; an I^2^-value above 50% indicates relevant heterogeneity [36].

To relate the size of the observed FMD responses to one or more potential covariates, we performed weighted meta-regression [37], [38]. The pre-defined covariates were health status (healthy or diseased), mean age, mean baseline FMD, dose of tea, type of tea (green or black tea), type of placebo (hot water or caffeine controlled), duration of tea (short-term/acute or long-term/chronic (>2 weeks)) and quality. In addition, we calculated a pooled FMD effect for different subgroups of the covariates under investigation. For the continuous variables, subgroups were defined based on above or below the median values. In case a subgroup consisted of less than 5 study arms, no subgroup analysis was performed; instead, we performed sensitivity analysis excluding those studies placed in the smallest subgroup.

To assess presence of publication bias, we visually inspected the symmetry of a funnel plot of the net effects on FMD observed in all studies expressed against their respective precisions (1/SE). The degree of funnel plot asymmetry was assessed by regression analysis of the standard normal deviate as a function of the precision [39]. Absence of publication bias is reflected in an intercept close to 0 with the slope of the regression line close to the overall effect size. The intercept provides a measure of asymmetry if P-value <0.1.

## Results

### Characteristics of included studies

**Figure 1:**
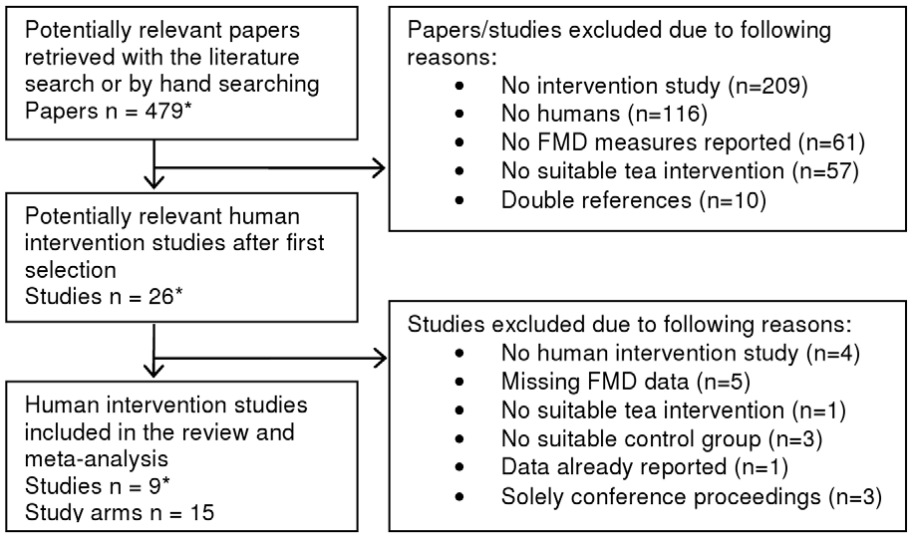
Flow diagram of the study selection procedure. *One study was identified after the systematic search through March 2009.

A total of 478 potentially relevant papers were retrieved with the systematic search. Based on the predefined selection criteria, 470 papers were excluded for different reasons (Figure 1). One eligible study was retrieved after the search [28], yielding a total of 9 studies with 15 study arms for this meta-analysis (Table 1). Seven studies had a crossover design [19], [22]–[24], [26], [40], [41] and two a parallel design [20], [28].

**Table 1:** Overview and characteristics of included studies.

		Subject characteristics				Treatment characteristics			%FMD outcomes			
Author and year^a^	Study design^b^	Total no of subjects^c^	Mean age (yr)	Gender (% male)	Health status^d^	Green or black	Dose of tea (mL/day)^e^	Duration^f^	Mean baseline (%)^g^	Net response (%)^h^	95% CI	Quality Score
Alexopoulos et al. 2008 [26]	R, PCW, SB, CO, dist	14	30	64	healthy	green	450	ST: 30 (/60/90) min	4.4	3.7	(0.7, 6.7)	5
Ardalan et al. 2007 [40]	NR, PCW, BNR, CO, prox	15	37.2	60	RTR	black	500	ST: 120 min	7.3	6.7	(4.4, 9.1)	4
Duffy et al. 2001 [19] (short-term)	R, PCW, SB, CO, prox	50	55	78	CAD	black	450	ST: 120 min	5.7	3.7	(2.6, 4.8)	6
Duffy et al. 2001 [19] (long-term)	R, PCW, SB, CO, prox	50	55	78	CAD	black	900	LT: 28 days	6.1	3.4	(2.3, 4.5)	6
Grassi et al. 2009 [41] (very low dose)	R, PCC, DB, CO, dist	19	32.9	100	healthy	black	120	ST upon LT: 7 days	7.8	1.2	(0.4, 2.0)	7
Grassi et al. 2009 [41] (low dose)	R, PCC, DB, CO, dist	19	32.9	100	healthy	black	240	ST upon LT: 7 days	7.8	1.3	(0.5, 2.1)	7
Grassi et al. 2009 [41] (medium dose)	R, PCC, DB, CO, dist	19	32.9	100	healthy	black	480	ST upon LT: 7 days	7.8	1.8	(1.2, 2.4)	7
Grassi et al. 2009 [41] (high dose)	R, PCC, DB, CO, dist	19	32.9	100	healthy	black	960	ST upon LT: 7 days	7.8	2.5	(2.0, 3.0)	7
Hodgson et al. 2002 [20]	R, PCW, SB, PA, dist	11/10	59.1	76	MHC	black	5×250	LT: 28 days	5.1^i^	2.3	(0.7, 3.9)	6
Hodgson et al. 2005 [22] (without meal)	R, PCW, SB, CO, dist	20	62.1	NA	CAD	black	3×250	ST: 60–90 min	4.3^i^	0.9	(−0.7, 2.5)	6
Hodgson et al. 2005 [22] (with meal)	R, PCW, SB, CO, dist	20	62.1	NA	CAD	black	3×250	ST: 60–90 min	5.4^i^	0.5	(−1.5, 2.5)	6
Jochmann et al. 2008 [23] (green tea)	RA, PCW, SB, CO, dist	21	58.7	0	healthy	green	500	ST: 120 min	6.4	3.8	(2.5, 5.1)	5.5
Jochmann et al. 2008 [23] (black tea)	RA, PCW, SB, CO, dist	21	58.7	0	healthy	black	500	ST: 120 min	6.4	2.7	(1.2, 4.2)	5.5
Lorenz et al. 2007 [24]	R, PCW, SB, CO, dist	16	59.5	0	healthy	black	500	ST: 120 min	6.9	2.2	(0.4, 4.0)	5.5
Park et al. 2009 [28]	R, PCW, OL, PA, dist	20/17	61.5	93	CKD	green	500	LT: 28 days	5.7	4.2	(2.1, 6.2)	5

a Duffy et al. [19] (both intervention arms) and Hodgson et al. [22] used a respective control period for each intervention period; Grassi et al. [41] and Jochmann et al. [23] used a single control period for all intervention periods. In case of multiple study arms, the intervention is specified between brackets.

b Study design is R (randomized), NR (not randomized), RA (randomization assumed), PCW (placebo controlled with water), PCC (placebo controlled with caffeine), DB (double blinded), SB (single blinded), BNR (blinding not reported), OL (open label), CO (crossover), PA (parallel), dist (distal occlusion), prox (proximal occlusion).

c For parallel studies: number in control group/number in treatment group.

d Health status is healthy, MHC (mildly hypercholesterolemic), CAD (coronary artery disease), RTR (renal transplant recipients), CKD (chronic kidney disease).

e The dosage of tea is expressed in mL tea per day. Only Grassi et al. [41] did not report the dosage of tea in such a unit and for that study, the volume of tea consumed was estimated from the concentration of flavonoids consumed per day.

f Duration is LT (long-term/chronic) or ST (short-term/acute).

g Mean baseline outcome (reference outcome) is the mean FMD at the end of the control period for crossover studies. For parallel studies, mean baseline outcome is the mean baseline FMD of the active treatment group. For Alexopoulos et al. [26], mean baseline outcome is the mean baseline FMD of the active treatment period (no endpoint FMD values reported).

h Net response was calculated by subtracting the mean FMD at the end of the active treatment period from the mean FMD at the end of the control period in case of crossover studies. For parallel studies, the mean FMD change from baseline in the control group was subtracted from the mean FMD change from baseline in the active treatment group.

NA not available.

i FMD measured with semi-automated edge-detection system.

A total of 213 subjects participated in the 9 studies. The number of subjects per study ranged from 14 to 50 subjects. Mean age ranged from 30.0 to 62.1 years and BMI from 22.1 to 29.7 kg/m^2^. One study included only men [41] whereas 2 studies included only women [23], [24]; in the remaining studies, except for 1 study that did not report gender [22], the percentage male ranged from 60% to 93%. In 5 out of 9 studies, subjects were healthy or mildly hypercholesterolemic; the other studies included renal transplant recipients [40], chronic kidney disease patients [28], or coronary artery disease patients [19], [22]. Mean baseline FMD ranged from 4.3% to 7.8%.

Seven studies investigated the effect of black tea; only 3 studies investigated (also) green tea [23], [26], [28]. In the majority of the studies, tea was brewed by infusing a certain amount of tea (5–10 g/d) in a defined amount of hot water (450–1250 mL) for a defined time period (2–5 min); in 2 studies [28], [41] tea was prepared by dissolving a tea powder in hot water. The study by Grassi et al. [41] used a control drink that contained an equal amount of caffeine as the tea interventions; the remaining studies used hot water without caffeine as control. Six studies investigated the acute effects on FMD, 30–120 minutes after drinking the prescribed amount of tea [19], [22]–[24], [26], [40], or the acute effects upon 1 week of daily tea consumption [41], whereas 3 studies (also) investigated the long-term effects (4 weeks) on FMD after an overnight fast and abstinence of tea [19], [20], [28]. Except for 2 studies that determined FMD with the occlusion cuff placed proximally to the area of measurement [19], [40], the majority of the studies determined FMD with the occlusion cuff placed distally. Only 2 studies [20], [22] used a semi-automated edge-detection system to determine the brachial artery diameter.

### Heterogeneity and publication bias

Between-study heterogeneity as assessed by the Q-statistic was significant (62.1, P-value <0.001, with accompanying I^2^ statistic 75.8%) justifying the use of a random effects model for calculating the pooled overall effect.

**Figure 2:**
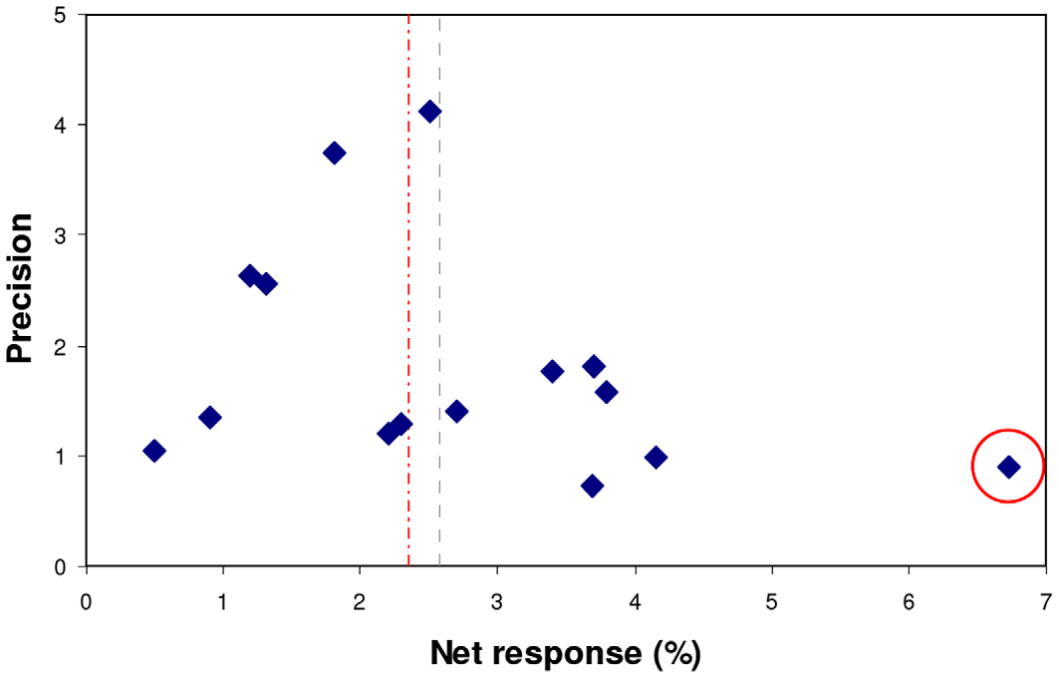
Funnel plot. The net FMD responses are expressed against their respective precisions (1/SE) in 15 study arms. The net responses are scattered around the pooled overall effect of 2.6% (dotted line). In case the most extreme outlier (red circle) is excluded from the analyses, the net responses are more symmetrically scattered around the adjusted pooled overall effect of 2.4% (red dotted line).

Visual inspection of the funnel plot (Figure 2) did not clearly indicate presence of publication bias. Indeed, regression analysis of the standard normal deviate as a function of the precision did not reveal clear funnel plot asymmetry (intercept: P-value  = 0.176), indicating absence of publication bias.

### Effect of tea on FMD

**Figure 3:**
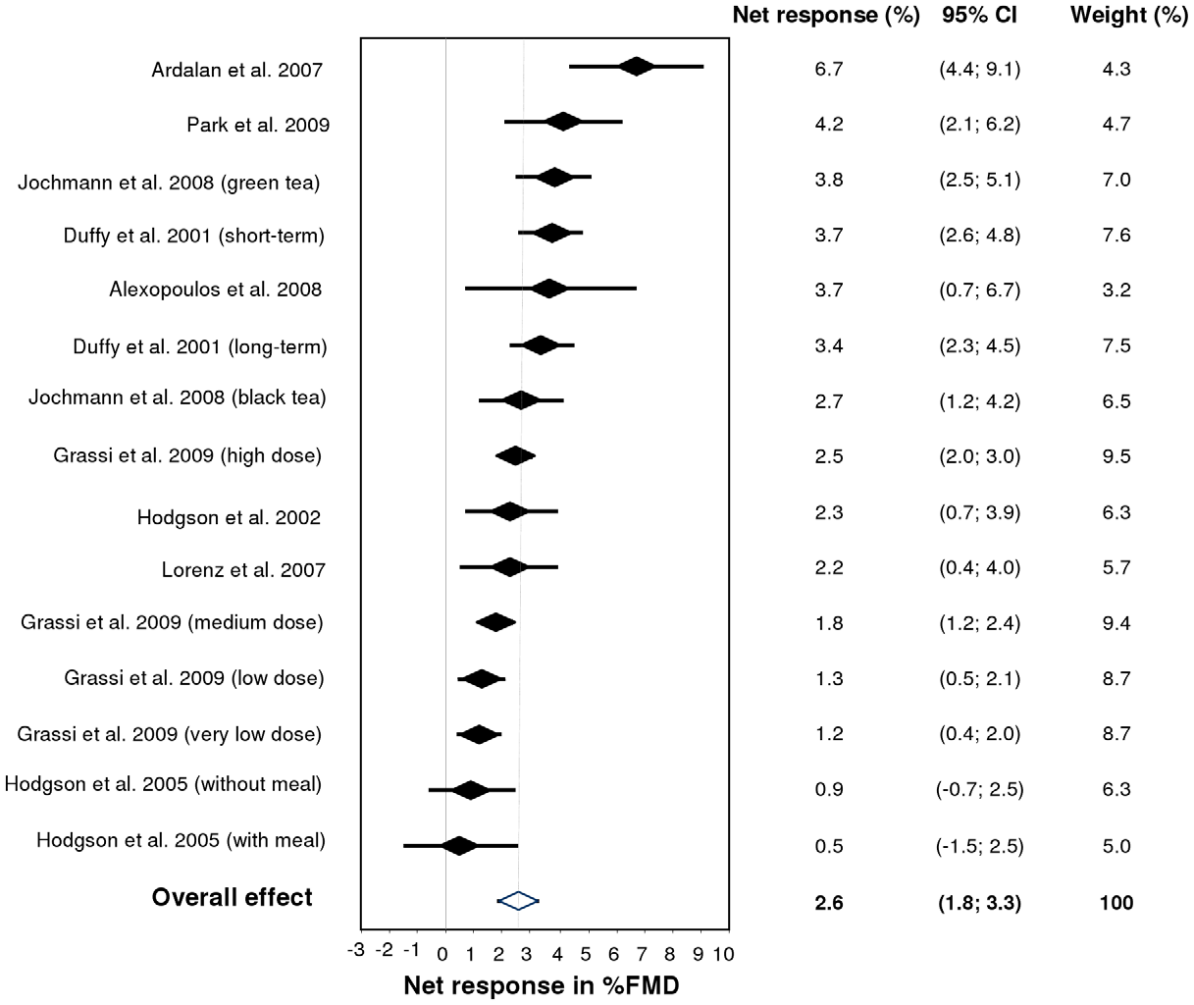
Forest plot. The net FMD responses and 95% confidence intervals of 15 study arms from 9 studies investigating the effect of tea on FMD are shown. The dotted line indicates the pooled overall FMD effect (2.6%), in which each study arm was weighed by the inverse of its variance (1/SE^2^). In case of multiple study arms, the intervention is specified between brackets.

In each of the individual studies, except for one [22], tea showed a statistically significant effect on FMD. In all studies combined, consumption of tea increased FMD vs. control by 2.6% of arterial dilation (95% CI: 1.8–3.3%; P-value <0.001), which is a relative improvement of approximately 40% compared to the average FMD of 6.3% under placebo (for cross-over trials) or baseline (for parallel trials) conditions (Figure 3). The median dose was 500 mL tea per day, which is equivalent to 2–3 cups per day.

### Covariate analysis

Of the variation in FMD responses between the studies, study quality score explained 52%, type of placebo 20%, type of tea 17%, health status 9%, study duration 6%, age 1%, baseline FMD 1%, and dose of tea <1%. Only the quality score of trials significantly correlated with the net FMD responses (P-value  = 0.002). However, this correlation lost significance (P-value  = 0.725) when all covariates were simultaneously included in the model. Furthermore, the study with the highest reported response in FMD was ranked with the lowest quality score [40]. Excluding this study from the analysis did not materially affect the overall effect (average FMD response 2.4%, see post-hoc analyses), but reduced the association between the quality score and the FMD effect considerably (P-value  = 0.030; R^2^  = 0.34). This indicates that the size of the response is less explained by the quality score when considering the disproportional impact of this study on the covariate analysis.

Analysis of the overall FMD effect of tea within predefined subgroups (with data from at least 5 study arms) indicated significant overall FMD effects for subgroups with diseased and healthy subjects, young and old subjects, high and low baseline FMD values, different amounts of tea prescribed, different study quality scores, black tea as intervention, when using hot water as control, and with acute intake of tea (Table 2). Between predefined subgroups, no significant differences in FMD responses were found, except for study quality score with higher quality studies showing smaller improvements in FMD (P-value  = 0.005).

**Table 2:** Effect of tea on FMD within and between different subgroups.

Trial characteristic	Stratification variable^a^	No of study arms	Pooled FMD effect (%)	95% CI	P-value
*Subgroup analysis*					
Health status	Diseased	6	3.1	(2.1; 4.2)	<0.001
	Healthy	9	2.3	(1.5; 3.0)	<0.001
	Δ	15	0.9	(−0.4; 2.2)	0.188
Baseline FMD	<6.4%	7	2.6	(1.6; 3.7)	<0.001
	≥6.4%	8	2.5	(1.7; 3.4)	<0.001
	Δ	15	0.1	(−1.2; 1.5)	0.866
Age	<55 yr	6	2.4	(1.4; 3.4)	<0.001
	≥55 yr	9	2.7	(1.8; 3.6)	<0.001
	Δ	15	−0.3	(−1.6; 1.1)	0.702
Dose	≤500 mL	10	2.9	(2.0; 3.7)	<0.001
	>500 mL	5	2.0	(0.9; 3.2)	<0.001
	Δ	15	0.8	(−0.6; 2.2)	0.259
Quality	Low quality (<6 points)	6	3.7	(2.7; 4.7)	<0.001
	High quality (≥6 points)	9	2.0	(1.4; 2.7)	<0.001
	Δ	15	1.7	(0.5; 2.9)	0.005
*Sensitivity analysis* b					
Type of tea	Black tea	12	2.3	(1.5; 3.1)	<0.001
Type of placebo	Water	11	3.0	(2.1; 4.0)	<0.001
Duration^c^	Short-term	8	3.0	(1.5; 4.4)	0.002
Cuff placement^d^	Distal	12	2.1	(1.5; 2.7)	<0.001

a For continuous variables, studies were divided in subgroups based on their medians: 6.4% for baseline FMD, 55 years for age, 500 mL for dose of tea, and 6.0 points for quality.

b For type of tea, type of placebo, study duration, and cuff placement, we performed sensitivity analysis due to limited number of studies (<5) in one of the subgroups.

c For the subgroup with short-term duration, we excluded the long-term study arms (>2 weeks) and the study arms that investigated acute upon longer-term effects.

d Post-hoc analysis.

In post hoc analyses, we checked the impact of 2 individual studies with specific characteristics on the overall estimated effect on FMD; we excluded the study by Grassi et al. [41] because it had with 4 study arms a disproportionally large weight, and the study by Ardalan et al. [40] because it included renal transplant recipients representing a very specific population. Exclusion of these studies did not substantially affect the outcome; the pooled overall FMD effect estimate without the study by Grassi et al. was 3.0% (95% CI: 2.1–4.0; P-value <0.001) and without the study by Ardalan et al. 2.4% (95% CI: 1.7–3.0; P-value <0.001) vs. the 2.6% (95% CI: 1.8–3.3%) in all 9 studies. Furthermore, excluding the most extreme outlier [40] from the publication bias analysis improved the symmetry of the funnel (intercept: P-value  = 0.401) supporting the indication of absence of publication bias (Figure 2).

Also in post hoc analyses, we investigated the impact of cuff position (either proximal or distal to the area of ultrasound measurement) on the FMD response to tea consumption. Based on meta-regression, cuff position was significantly correlated with the FMD responses; smaller responses to tea were observed with distal occlusion (P-value 0.017; R^2^ = 0.36). Due to the limited number of studies that used proximal occlusion, we performed sensitivity analysis only including those studies that used distal occlusion (n = 12) and calculated a significant overall FMD effect of 2.1% (95% CI: 1.5–2.7; P-value <0.001).

### Tea effects on endothelium-independent vasodilation

The majority of studies, except for two [40], [41], assessed endothelium-independent vasodilation by sublingual administration of nitroglycerine spray. No effect of tea was found in 6 out of 7 studies (data not shown). Only Hodgson et al. [20] reported a small but significant enhancement of the endothelium-independent vasodilation after black tea consumption.

## Discussion

In this meta-analysis, we summarized published evidence from 9 human intervention studies that investigated the effect of tea consumption on endothelial function as measured by FMD. It was found that moderate consumption of tea substantially enhances FMD. This is in line with findings from a previous meta-analysis on flavonoids/flavonoid-rich foods and cardiovascular risk factors [27], that included 2 studies on tea and FMD published at that time [19], [22]. The effect of tea on FMD seems robust because the estimated overall effect is large and the effect was observed in 8 out of 9 studies including different study populations and different tea types. Also, we could not detect indications of systematic publication bias.

During the last decade, FMD has been increasingly used as tool to assess effects of therapeutic interventions on endothelial function in humans. The relevance of FMD for predicting CVD risk independent of other well-established risk factors is crucial in this respect. Several prospective studies, but not all [10]–[12], indicate an independent inverse association between FMD and risk for cardiovascular events, not only in patients with varying stages of arterial disease [13]–[16], but also in subjects without diagnosis of CVD [17], [18]. FMD has been shown to add to the predictive value of ankle-brachial pressure index [14] and hyperaemic flow velocity [42] in patients with peripheral arterial disease. In addition, Chan et al. [16] showed an interaction between carotid plaque burden and endothelial function for predicting future adverse vascular events in coronary artery disease patients. On the contrary, FMD does not seem to be independently associated with CVD outcomes when intima-media thickness is considered at the same time [11], [12]. Whether improvement in FMD in response to treatment can predict CVD risk was assessed in at least 2 studies. The study by Kitta et al. [43] suggested that optimized therapy to reduce risk factors for coronary artery disease is effective in improving FMD in CVD patients, with persistent impairment in FMD being an independent predictor of events. Modena et al. [44] showed that in postmenopausal women, antihypertensive therapy positively affects FMD, and that improvement in FMD identified patients with a subsequent more favourable prognosis for events when adjusted for changes in other risk factors such as SBP and DBP. Thus, taken together, an independent association is suggested between FMD and CVD risk, although causality remains to be proven.

Potential health properties of tea are likely due to certain chemical substances extracted from the tea leaves. The active tea substances responsible for the increase in FMD are still unknown, but a specific role for certain flavonoids, such as the catechins epicatechin, epigallocatechin, epigallocatechin gallate, and epicatechin gallate has been suggested [45]. The caffeine in tea probably does not contribute to the effect; an oral dose of pure caffeine was found to not significantly affect FMD [19] or even reduced FMD [46]. Also, tea solids dose-dependently affect FMD when caffeine intake is kept constant [41]. In one study [22], tea did not significantly improve FMD. An explanation may be found in the fact that FMD was measured only shortly (60–90 min) after tea consumption. Since catechin plasma concentrations peak 1–2 h after intake in fasted state and 2–3 h in postprandial state [47], [48], it may be that the catechins were not yet fully absorbed at the time of the FMD assessment. However, whether the catechins are the true actives remains to be elucidated.

With the studies included in our analysis, we were not able to confirm a dose-response relation between amount of tea consumed and FMD response as was suggested by the study by Grassi et al. [41]. The absence of a clear dose-response relation may be due to the inaccuracy of expressing tea intake as volume of beverage consumed per day rather than as amount of potentially active substances ingested in the different tea interventions. The included studies used different tea products and preparation methods with varying amounts of tea leaves, brewing time, and water temperatures. These differences presumably result in different concentrations of active substances per tea serving [49]. It has been estimated that approximately 84% of total polyphenol content in tea is flavonoids [50]. Black tea contains on average 992 mg/L of total polyphenols, whereas green tea contains 591 mg/L [49]. A dose of 500 mL of tea (∼2–3 cups) can thus be estimated to contain on average 415 mg of flavonoids for black tea and 248 mg for green tea. However, these are rough estimates and, in practice, the variation between tea brews will be large.

A proposed mechanism by which dietary flavonoids could affect FMD is that they improve the bioactivity of the endothelium-derived vasodilator NO [51] by enhancing NO synthesis or by decreasing superoxide-mediated NO breakdown [52]. Flavonoids may increase endothelial NO production [53], [54] by stimulating Akt-mediated endothelial-derived NO synthase activity [55], [56], and additionally decrease levels of the vasoconstrictor endothelin-1 [53]. Another mechanistic explanation is that methylated flavonoids inhibit nicotinamide adenine dinucleotide phosphate oxidase activity, and thereby reduce the generation of reactive superoxide and hydroxyperoxide [57]. However, the precise mechanism is not yet fully revealed, and different potentially active flavonoids and their metabolites may have different effects. No indication of endothelial-independent vasodilatory effects of tea was found in the included studies. Although not conclusive, this makes it unlikely that consumption of tea sensitizes arterial smooth muscle cells for NO.

Because heterogeneity between studies was significant, we analyzed the impact of potential covariates on the relationship between tea and FMD in order to identify factors that could explain differences in findings between studies. Only the study quality score was significantly associated with the size of the FMD response observed after tea consumption, with higher quality studies showing smaller improvements in FMD than the lower quality studies. However, this association was no longer significant when corrected for other covariates in the same model. It should be noted that scoring the quality of a study is intrinsically subjective. Thus, the apparent relation between study quality and observed effect on FMD should be interpreted with caution. Overall, the number of studies included in this meta-analysis was too limited to allow a thorough, reliable analysis of sources of heterogeneity.

Our study has other limitations. Firstly, reproducibility of a functional marker such as FMD is low as compared to most biochemical markers. Standardized protocols for imaging techniques, as for example recently described by Thijsen et al. [58], are required to reduce within-subject variability. These should include multiple measurements and duplicate readings, preferably using automated vessel wall boundary detection devices [59], [60]. Such an automated device was used in only 2 out of 9 studies included in the present meta-analysis. Also, the position of the occlusion cuff, either proximal or distal to the area of FMD measurement, is important to consider. A post-hoc analysis suggested that cuff position has an impact on the size of the FMD response after tea consumption, with larger effects on FMD when using the proximal occlusion method, which may explain part of the heterogeneity found between the studies. Doshi et al. [61] have shown that FMD assessed by the distal occlusion method can be abolished by infusion of the NO synthase inhibitor NG-monomethyl-L-arginine, but is only partly inhibited (by ∼35%) when using the proximal occlusion method. This indicates that dilation after proximal occlusion is not entirely NO-mediated. Because an enhanced tea effect on FMD was found with the proximal cuff placement, it may be speculated that tea affects the vasodilatory response beyond affecting NO bioavailability via mechanisms still to be revealed [58], [62]. A second limitation of our analysis is that the majority of included studies measured FMD acutely, i.e. about 2 hours after ingestion of a defined dose of tea. Although an improvement in FMD by tea was also seen after longer-term (4 weeks) regular tea consumption, the clinical relevance of acute improvements in FMD is unclear. Thirdly, this meta-analysis included 2 studies that compared more than 1 active treatment to the same control treatment [23], [41]. Although bias due to multiple inclusion of the same control group can not be excluded, we considered this as the best possible approach to not exclude valuable data from treatment arms (i.e. different doses). Excluding the study that contributed most to the overall estimate with 4 treatment arms vs. the same control group [41] did not materially affect the results.

In conclusion, our findings indicate that tea consumption results in substantial effects on the vascular endothelium as indicated by an improved endothelial-dependent vasodilation in the first hours after intake, and also after longer-term regular consumption of tea. This effect may partly explain the relationship between tea consumption and reduced CVD risk in population studies when assuming that FMD offers independent predictive value for CVD endpoints. However, to what extent tea-mediated improvement in endothelial function is indeed causally related to a reduction in cardiovascular events can only be determined by large, long-term randomized trials on clinical endpoints.

## Supporting Information

Checklist S1 **Prisma checklist.**
(DOC)
